# Lossy Compression of Individual Sequences Revisited: Fundamental Limits of Finite-State Encoders

**DOI:** 10.3390/e26020116

**Published:** 2024-01-28

**Authors:** Neri Merhav

**Affiliations:** The Viterbi Faculty of Electrical and Computer Engineering, Technion-Israel Institute of Technology, Technion City, Haifa 3200003, Israel; merhav@technion.ac.il; Tel.: +972-4-8294737

**Keywords:** rate-distortion, source coding, finite-state encoders, random coding, code ensemble, lossy compression, universal coding, universal distribution, LZ algorithm

## Abstract

We extend Ziv and Lempel’s model of finite-state encoders to the realm of lossy compression of individual sequences. In particular, the model of the encoder includes a finite-state reconstruction codebook followed by an information lossless finite-state encoder that compresses the reconstruction codeword with no additional distortion. We first derive two different lower bounds to the compression ratio, which depend on the number of states of the lossless encoder. Both bounds are asymptotically achievable by conceptually simple coding schemes. We then show that when the number of states of the lossless encoder is large enough in terms of the reconstruction block length, the performance can be improved, sometimes significantly so. In particular, the improved performance is achievable using a random-coding ensemble that is universal, not only in terms of the source sequence but also in terms of the distortion measure.

## 1. Introduction

**Figure 1:**
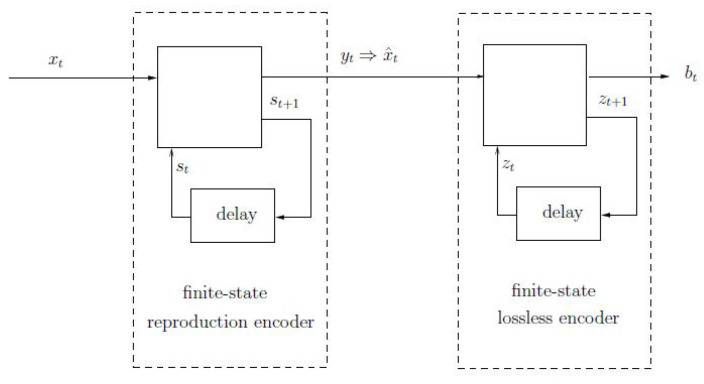
Finite-state reproduction encoder followed by a finite-state lossless encoder.

We revisit the classical domain of rate-distortion coding applied to finite-alphabet sequences, focusing on a prescribed distortion function [1,2], (Chapter 10), [3] (Chapter 9), [4,5], (Chapters 7, 8). Specifically, our attention is directed toward encoders comprising finite-state reproduction encoders followed by information-lossless finite-state encoders that compress reproduction sequences without introducing additional distortion (see Figure 1). In essence, our principal findings are in establishing two asymptotically achievable lower bounds for the optimal compression ratio of an individual source sequence of length *n*, utilizing any finite-state encoder with the aforementioned structure, where the lossless encoder possesses *q* states. These lower bounds can both be conceptualized as the individual-sequence counterparts to the rate-distortion function of the given source sequence, akin to the lossless finite-state compressibility of a source sequence serving as the individual-sequence analog of entropy. However, before delving into the intricacies of our results, a brief overview of the background is warranted.

Over the past several decades, numerous research endeavors have been spurred by the realization that source statistics are seldom, if ever, known in practical scenarios. Consequently, these efforts have been dedicated to the pursuit of universal coding strategies that remain independent of unknown statistics while asymptotically approaching lower bounds, such as entropy in lossless compression or the rate-distortion function in the case of lossy compression, as the block length extends indefinitely. Here, we offer a succinct and non-exhaustive overview of some pertinent earlier works.

In the realm of lossless compression, the field of universal source coding has achieved a high level of sophistication and maturity. Davisson’s seminal work [6] on universal-coding redundancies has introduced the pivotal concepts of weak universality and strong universality, characterized by vanishing maximin and minimax redundancies, respectively. This work has also elucidated the link between these notions and the capacity of the ’channel’ defined by the family of conditional distributions of the data to be compressed, given the index or parameter of the source in the class [7,8,9]. For numerous parametric source classes encountered in practice, the minimum achievable redundancy of universal codes is well-established as being dominated by $\frac{k\;\log\;n}{2n}$, where *k* denotes the number of degrees of freedom of the parameter, and *n* is the block length [10,11,12,13]. Davisson’s theory gives rise to a central idea of constructing a Shannon code based on the probability distribution of the data vector with respect to a mixture, incorporating a certain prior function, of all sources within the class. Rissanen, credited with the invention of the minimum description length (MDL) principle [14], established a converse to a coding theorem in [15]. This theorem asserts that, asymptotically, no universal code can achieve redundancy below $\left(1-\epsilon \right)\frac{k\;\log\;n}{2n}$, with a possible exception of sources from a subset of the parameter space, the volume of which diminishes as n→∞ for every positive ϵ. Merhav and Feder [16] generalized this result to more extensive classes of sources, substituting the term $\frac{k\;\log\;n}{2n}$ with the capacity of the aforementioned ’channel’. Subsequent studies have further refined redundancy analyses and contributed to ongoing developments in the field.

In the broader domain of universal lossy compression, the theoretical landscape is regrettably not as sharply defined and well-developed as in the lossless counterpart. In this study, we narrow our focus to a specific class known as *d*-semifaithful codes [17] codes that fulfill the distortion requirement with probability one. Zhang, Yang, and Wei [18] have demonstrated a notable contrast with lossless compression, establishing that, even when source statistics are perfectly known, achieving redundancy below $\frac{\log\;n}{2n}$ in the lossy case is impossible, although $\frac{\log\;n}{n}$ is attainable. The absence of source knowledge imposes a cost in terms of enlarging the multiplicative constant associated with $\frac{\log\;n}{n}$. Yu and Speed [19] established weak universality, introducing a constant that grows with the cardinalities of the source and reconstruction alphabets [20]. Ornstein and Shields [17] delved into universal *d*-semifaithful coding for stationary and ergodic sources concerning the Hamming distortion measure, demonstrating convergence to the rate-distortion function with the probability one. Kontoyiannis [21] made several noteworthy contributions: Firstly, a central limit theorem (CLT) with a $O(1/\sqrt{n})$ redundancy term, featuring a limiting Gaussian random variable with constant variance. Secondly, the law of iterated logarithm (LIL) with redundancy proportional to $\sqrt{\log(\log n)/n}$ infinitely often with probability one. A counterintuitive conclusion from [21] is the priceless nature of universality under these CLT and LIL criteria. In [22], optimal compression is characterized by the negative logarithm of the probability of a sphere of radius nD around the source vector with respect to the distortion measure, where *D* denotes the allowed per-letter distortion. The article also introduces the concept of a random coding ensemble with a probability distribution given by a mixture of all distributions in a specific class. In two recent articles, Mahmood and Wagner [23,24] delved into the study of *d*-semifaithful codes that are strongly universal concerning both the source and the distortion function. The redundancy rates in [23] behave like $\frac{\log\;n}{n}$ but with different multiplicative constants. Other illuminating results regarding a special distortion measure are found in [25].

A parallel path of research in the field of universal lossless and lossy compression, spearheaded by Ziv, revolves around the individual-sequence approach. In this paradigm, no assumptions are made about the statistical properties of the source. The source sequence to be compressed is treated as an arbitrary deterministic (individual) sequence, but instead, limitations are imposed on the implementability of the encoder and/or decoder using finite-state machines. This approach notably encompasses the widely celebrated Lempel–Ziv (LZ) algorithm [26,27,28], along with subsequent advancements broadening its scope to both lossy compression with and without side information [29,30], as well as joint source-channel coding [31,32]. In the lossless context, the work in [33] establishes an individual-sequence analog akin to Rissanen’s result, where the expression $\frac{k\;\log\;n}{2n}$ continues to denote the best achievable redundancy. However, the primary term in the compression ratio is the empirical entropy of the source vector, deviating from the conventional entropy in the probabilistic setting. The converse bound presented in [33] is applicable to the vast majority of source sequences within each type, echoing the analogy with Rissanen’s framework concerning the majority of the parameter space. It is noteworthy that this converse result retains a semblance of the probabilistic setting, as asserting the relatively small number of exceptional typical sequences is equivalent to assuming a uniform distribution across the type and asserting a low probability of violating the bound. Conversely, the achievability result in [33] holds pointwise for every sequence. A similar observation applies to [34], where asymptotically pointwise lossy compression was established concerning first-order statistics (i.e., “memoryless” statistics), emphasizing distortion-universality, akin to the focus in [23,24]. A similar fusion of the individual-sequence setting and the probabilistic framework is evident in [35] concerning universal rate-distortion coding. However, akin to the approach in [34], there is no constraint on finite-state encoders/decoders as in [33]. Notably, the converse theorem in [35] states that for any variable-rate code and any distortion function within a broad class, the vast *majority* of reproduction vectors representing source sequences of a given type (of any fixed order) must exhibit a code length essentially no smaller than the negative logarithm of the probability of a ball with a normalized radius *D* (where *D* denotes the allowed per-letter distortion). This ball is centered at the specified source sequence, and the probability is computed with respect to a universal distribution proportional to $2^{-LZ(\hat{x})}$, where $LZ(\hat{x})$ denotes the code length of the LZ encoding of the reproduction vector $\hat{x}$.

The emphasis on the term “majority” in the preceding paragraph, as highlighted earlier, necessitates clarification. It should be noted that in the absence of constraints on encoding memory resources, such as the finite-state machine model mentioned earlier, there cannot exist any meaningful lower bound that universally applies to every individual sequence. The rationale is straightforward: for any specific individual source sequence, it is always possible to devise an encoder compressing that sequence to a single bit (even losslessly). For instance, by designating the bit ‘0’ as the compressed representation of the given sequence and appending the bit ‘1’ as a header to the uncompressed binary representation of any other source sequence. In this scenario, the compression ratio for the given individual sequence would be 1/n, dwindling to zero as *n* grows indefinitely. Therefore, it is clear that any non-trivial lower bound that universally applies to *every* individual source sequence at the same time necessitates reference to a class of encoders/decoders equipped with constrained resources, such as those featuring a finite number of states.

In this work, we consider lossy compression of individual source sequences using finite-state encoders whose structure is as follows: Owing to the fact that, without loss of optimality, every lossy encoder can be represented as a cascade of a reproduction encoder and a lossless (or “noiseless”) encoder (see, e.g., [36], particularly the discussion around Figure 1), we consider a class of lossy encoders that can be implemented as a cascade of a finite-state reproduction encoder and a finite-state lossless encoder; see Figure 1. The finite-state reproduction encoder model is a generalization of the well-known finite-state vector quantizer (FSVQ), see, e.g., [37,38] (Chapter 14). It is designed to produce reproduction vectors of dimension *k* in response to source vectors of dimension *k*, while complying with the distortion constraint for every such vector. The finite-state lossless encoder is the same as in [27]. The number of states of the reproduction encoder can be assumed to be very large (large enough to store many recent input blocks). Both the dimension, *k*, and the number of states, *q*, of the lossless encoder are assumed to be small compared to the total length, *n*, of the source sequence to be compressed, similar to [27] (and other related works), where the regime q≪n is also assumed.

One of our main messages in this work is that the relationship between *q* and *k* is important, not only with how they both relate to *n*. If *q* is large in terms of *k*, one can do much better than if it is small. Accordingly, we first derive two different lower bounds to the compression ratio under the assumption that q≪k, which are both asymptotically achievable by conceptually simple schemes that, within each *k*-block, seek the most compressible *k*-vector within a ball of ‘radius’ kD around the source block. The motivation for deriving two different bounds is that each one of them has its own strengths and it is not apparent that any one of them always dominates the other (see the details in the sequel, and in particular, the third paragraph of the discussion in Section 3). We compare the performance of the achievability scheme to the ensemble performance of a universal coding scheme that can be implemented when *q* is exponential in *k*. The improvement can sometimes be considerably large. The universality of the coding scheme is two-fold: both in the source sequence to be compressed and in the distortion measure in the sense that the order of codewords within the typical codebook (which affects the encoding of their indices) is asymptotically optimal no matter which distortion measure is used (see [35] for a discussion of this property). The intuition behind this improvement is that when *q* is exponential in *k*, the memory of the lossless encoder is large enough to store the entire input blocks and thereby exploit the sparseness of the reproduction codebook in the space of *k*-dimensional vectors with components in the reproduction alphabet. The asymptotic achievability of the lower bound will rely on the direct coding theorem of [35].

Bounds on both lossless and lossy compression of individual sequences using finite-state encoders and decoders have been explored in previous works, necessitating a contextualization of the present work. As previously mentioned, the cases of (almost) lossless compression were examined in [26,27,30]. In [32], the lossy case was considered, incorporating both a finite-state encoder and a finite-state decoder in the defined model. However, in the proof of the converse part, the assumption of a finite-state encoder was not essential; only a finite number of states of the decoder was required. In a subsequent work, [31], the finite number of states for both the encoder and decoder were indeed utilized. This holds true for [29] as well, where the individual-sequence analog of the Wyner–Ziv problem was investigated with more restrictive assumptions on the structure of the finite-state encoder. In contrast, the current work restricts only the encoder to be a finite-state machine, presenting a natural generalization of [27] to the lossy case. Specifically, one of our achievable lower bounds can be regarded as an extension of the compressibility bound found in [27] Corollary 1 to the lossy scenario. It is crucial to note that, particularly in the lossy case, it is more imperative to impose limitations on the encoder than the decoder, as encoding complexity serves as the practical bottleneck. Conversely, for deriving converse bounds, it is stronger and more general not to impose any constraints on the decoder.

The outline of this paper is as follows. In Section 2, we establish notation, as well as definitions, and spell out the objectives. In Section 3, we derive the main results and discuss them. Finally, in Section 4, we summarize the main contributions of this work and make some concluding remarks.

## 2. Notation, Definitions, and Objectives

Throughout the paper, random variables will be denoted by capital letters; specific values they may take will be denoted by the corresponding lowercase letters, and their alphabets will be denoted by calligraphic letters. Random vectors and their realizations will be denoted, respectively, by capital letters and the corresponding lowercase letters, both in the boldface font. Their alphabets will be superscript by their dimensions. The source vector of length *n*, $\left(x_{1},x_{2},\ldots ,x_{n}\right)$, with components, $x_{i}$, i=1,2,…,n, from a finite alphabet, X, will be denoted by $x^{n}$. The set of all such *n*-vectors will be denoted by $X^{n}$, which is the *n*-th order Cartesian power of X. Likewise, a reproduction vector of length *n*, $\left({\hat{x}}_{1},\ldots ,{\hat{x}}_{n}\right)$, with components, ${\hat{x}}_{i}$, i=1,…,n, from a finite alphabet, $\hat{X}$, will be denoted by ${\hat{x}}^{n}\in {\hat{X}}^{n}$. The notation ${\hat{X}}^{*}$ will be used to designate the set of all finite-length strings of symbols from $\hat{X}$.

For i≤j, the notation $x_{i}^{j}$ will be used to denote the substring $\left(x_{i},x_{i+1},\ldots ,x_{j}\right)$. For i=1, subscript ‘1’ will be omitted, and so, the shorthand notation of $\left(x_{1},x_{2},\ldots ,x_{n}\right)$ will be $x^{n}$. Similar conventions will apply to other sequences. Probability distributions will be denoted by the letter *P* or *Q* with possible subscripts, depending on the context. The probability of an event A will be denoted by Pr{A}, and the expectation operator with respect to (w.r.t.) a probability distribution *P* will be denoted by E{·}. The logarithmic function, logx, will be understood to refer to base 2. Logarithms to base *e* will be denoted by ln. Let $d:X\times \hat{X}\to R$ be a given distortion function between source symbols and reproduction symbols. The distortion between vectors will be defined additively as $d\left(x^{n},{\hat{x}}^{n}\right)=\sum_{i=1}^{n}d\left(x_{i},{\hat{x}}_{i}\right)$ for every positive integer, *n*, and every $x^{n}\in X^{n}$, ${\hat{x}}^{n}\in {\hat{X}}^{n}$.

Consider the encoder model depicted in Figure 1, which is a cascade of a finite-state reproduction encoder (FSRE) and a finite-state lossless encoder (FSLE). This encoder is fully determined by the set $E=(X,\hat{X},S,Z,u,v,f,g,k)$, where X is the source input alphabet of size α, $\hat{X}$ is the reproduction alphabet of size β, S is a set of FSRE states, Z is a set of FSLE states of size *q*, *u*, and *v* are functions that define the FSRE, *f* and *g* are functions that define the FSLE (both to be defined shortly), and *k* is a positive integer that designates the basic block length within which the distortion constraint must be kept, as will be described shortly. The number of states, |S|, of the FSRE may be assumed arbitrarily large (as the lower bounds to be derived will actually be independent of this number). In particular, it can be assumed to be large enough to store several recent input *k*-blocks.

According to this encoder model, the input, $x_{t}\in X$, t=1,2,…, is fed sequentially into the FSRE, which goes through a sequence of states $s_{t}\in S$, and produces an output sequence, $y_{t}\in {\hat{X}}^{*}$ of variable-length strings of symbols from $\hat{X}$, with the possible inclusion of the empty symbol, λ, of length zero. Referring to Figure 1, the FSRE is defined by the recursive equations:

(1) $$\begin{array}{ccc} y_{t} & = & u(x_{t},s_{t}) \end{array}$$

(2) $$\begin{array}{ccc} s_{t+1} & = & v(x_{t},s_{t}), \end{array}$$

for t=1,2,…, where the initial state, $s_{1}$, is assumed to be some fixed member of S.

**Remark** **1.** *The above-defined model of the FSRE has some resemblance to the well-known model of the finite-state vector quantizer (FSVQ) [37], [38] (Chapter 14), but it is in fact, considerably more general than the FSVQ. Specifically, the FSVQ works as follows. At each time instant t, it receives a source vector $x_{t}$ and outputs a finite-alphabet variable, $u_{t}$, while updating its internal state, $s_{t}$. The encoding function is $u_{t}=a\left(x_{t},s_{t}\right)$ and the next-state function is $s_{t+1}=\phi \left(u_{t},s_{t}\right)$. Note that the state evolves in response to $\left\{u_{t}\right\}$ (and not $x_{t}$), so that the decoder will be able to maintain its own copy of $\left\{s_{t}\right\}$. At the decoder, the reproduction is generated according to ${\hat{x}}_{t}=b\left(u_{t},s_{t}\right)$, and the state is updated again using $s_{t+1}=\phi \left(u_{t},s_{t}\right)$. By cascading the FSVQ encoder and its decoder, one obtains a system with input $x_{t}$ and output ${\hat{x}}_{t}$, which is basically a special case of our FSRE with the functions u and v being given by u(x,s)=b(a(x,s),s) and v(x,s)=ϕ(a(x,s),s).*

As described above, given an input block of length *k*, $\left(x_{1},x_{2},\ldots ,x_{k}\right)$, the FSRE generates a corresponding output block, $\left(y_{1},y_{2},\ldots ,y_{k}\right)$, while traversing a sequence of states $\left(s_{1},\ldots ,s_{n}\right)$. The FSRE must be designed in such a way that the total length of the concatenation of the (non-empty) variable-length strings, $y_{1},y_{2},\ldots ,y_{k}$, is equal to *k* as well. Accordingly, given $\left(y_{1},y_{2},\ldots ,y_{k}\right)$, let $\left({\hat{x}}_{1},{\hat{x}}_{2},\ldots ,{\hat{x}}_{k}\right)$ denote the corresponding vector of reproduction symbols from $\hat{X}$, which forms the output of the FSRE. This formal transformation from $y^{k}$ to ${\hat{x}}^{k}$ is designated by the expression $y_{t}\Rightarrow {\hat{x}}_{t}$ in Figure 1.

**Example** **1.** *Let $X=\hat{X}=\left\{a,b,c\right\}$, and suppose that the FSRE is a block code of length k=5. Suppose also that $x^{5}=\left(a,a,b,c,c\right)$ and $y^{5}=\left(\lambda ,\lambda ,\lambda ,\lambda ,‘aabb’\right)$. Then, ${\hat{x}}^{5}=\left(a,a,b,b,c\right)$. The current state, in this case, is simply the contents of the input, starting from the beginning of the current block and ending at the current input symbol. Accordingly, the encoder idles until the end of the input block, and then it produces the full output block.*

The parameter *k* of the encoder *E* is the length of the basic block that is associated with the distortion constraint. For a given input alphabet X, reconstruction alphabet $\hat{X}$, and distortion function *d*, we denote by E(q,k,D) the class of all finite-state encoders with the above-described structure; in this class, the number of FSLE states is *q*, the dimension of the FSRE is *k*, and $d(x^{k},{\hat{x}}^{k})\leq kD$ for every above-described $x^{k}\in X^{k}$. For future use, we also define the ‘ball’

(3) $$B\left(x^{k},D\right)=\left\{{\hat{x}}^{k}:\;d\left(x^{k},{\hat{x}}^{k}\right)\leq kD\right\}.$$

**Remark** **2.** *Note that the role of the state variable, $s_{t}$, might not be only to store information from the past of the input, but possibly also to maintain the distortion budget within each k-block. At each time instant, t, the state can be used to update the remaining distortion allowed until the end of the current k-block. For example, if the entire allowed distortion budget, kD, has already been exhausted before the current k-block has ended, then in the remaining part of the current block, the encoder must carry on losslessly, that is, it must produce reproduction symbols that incur zero distortion relative to the corresponding source symbols.*

The FSLE is defined similarly to the description provided in [27]. Specifically, the output of the FSRE, ${\hat{x}}_{t}\in \hat{X}$, t=1,2,…, is fed sequentially into the FSLE, which in turn goes through a sequence of states $z_{t}\in Z$, and produces an output sequence, $b_{t}\in {\left\{0,1\right\}}^{*}$ of variable-length binary strings, with the possible inclusion of the empty symbol, λ, of length zero. Accordingly, the FSLE implements the recursive equations,

(4) $$\begin{array}{ccc} b_{t} & = & f({\hat{x}}_{t},z_{t}) \end{array}$$

(5) $$\begin{array}{ccc} z_{t+1} & = & g({\hat{x}}_{t},z_{t}), \end{array}$$

for t=1,2,…, where the initial state, $z_{1}$, is assumed to be some fixed member of Z.

With a slight abuse of notation, we adopt the extended use of encoder functions *u*, *v*, *f*, and *g*, to designate output sequences and final states, which result from the corresponding initial states and inputs. We use the notations $u(s_{1},x^{n})$, $v(s_{1},x^{n})$, $f(z_{1},u\left(s_{1},x^{n}\right))$, and $g(z_{1},u\left(s_{1},x^{n}\right))$ for ${\hat{x}}^{n}$, $s_{n+1}$, $b^{n}$, and $z_{n+1}$, respectively. We assume the FSLE to be information lossless, and define it similarly to the description provided in [27], as follows. For every $(z_{1},s_{1})\in Z\times S$, every positive integer *n*, and every $x^{n}\in X^{n}$, the triple $\left(z_{1},f\left(z_{1},u\left(s_{1},x^{n}\right)\right),g\left(z_{1},u\left(s_{1},x^{n}\right)\right)\right)$ uniquely determines ${\hat{x}}^{n}$.

Given an encoder $E=\left(X,\hat{X},S,Z,u,v,f,g,k\right)\in E\left(q,k,D\right)$, and a source string $x^{n}$, where *n* is divisible by *k*, the compression ratio of $x^{n}$ by E is defined as

(6) $$\rho \left(x^{n};E\right)=\frac{L(b^{n})}{n},$$

where $L\left(b^{n}\right)=\sum_{t=1}^{n}\ell \left(b_{t}\right)$, $\ell (b_{t})$ being the length (in bits) of the binary string $b_{t}$. Next, define

(7) $$\rho \left(x^{n};E\left(q,k,D\right)\right)=\min_{E\in E(q,k,D)}\rho \left(x^{n};E\right).$$

Our main objective is to derive bounds for $\rho (x^{n};E\left(q,k,D\right))$ for large *k* and n≫k, with special interest in the case where *q* is large enough (in terms of *k*), but still fixed and independent of *n*, so that the FSLE could take advantage of the fact that not necessarily every ${\hat{x}}^{n}\in {\hat{X}}^{n}$ can be obtained as an output of the given FSRE. In particular, a good FSLE with long memory should exploit the sparseness of the reproduction codebook relative to the entire space of *k*-vectors in ${\hat{X}}^{k}$.

## 3. Lower Bounds

To present both the lower bounds and the achievability, we briefly review a few terms and facts concerning the 1978 version of the Lempel–Ziv algorithm (a.k.a. the LZ78 algorithm) [27]. The incremental parsing procedure of the LZ78 algorithm is a procedure of sequentially parsing a vector, ${\hat{x}}^{k}\in {\hat{X}}^{k}$, such that each new phrase is the shortest string that has not been encountered before as a parsed phrase, with the possible exception of the last phrase, which might be incomplete. For example, the incremental parsing of the vector ${\hat{x}}^{15}=abbabaabbaaabaa$ is a,b,ba,baa,bb,aa,ab,aa. Let $c({\hat{x}}^{k})$ denote the number of phrases in ${\hat{x}}^{k}$ resulting from the incremental parsing procedure (in the above example, $c({\hat{x}}^{15})=8$). Let $LZ({\hat{x}}^{k})$ denote the length of the LZ78 binary compressed code for ${\hat{x}}^{k}$. According to [27] Theorem 2,

(8) $$\begin{array}{ccc} LZ({\hat{x}}^{k}) & \leq & \left[c\left({\hat{x}}^{k}\right)+1\right]\log\left\{2\beta \left[c\left({\hat{x}}^{k}\right)+1\right]\right\}\\ & = & c\left({\hat{x}}^{k}\right)\log\left[c\left({\hat{x}}^{k}\right)+1\right]+c\left({\hat{x}}^{k}\right)\log\left(2\beta \right)+\log\left\{2\beta \left[c\left({\hat{x}}^{k}\right)+1\right]\right\}\\ & = & c\left({\hat{x}}^{k}\right)\log c\left({\hat{x}}^{k}\right)+c\left({\hat{x}}^{k}\right)\log\left[1+\frac{1}{c({\hat{x}}^{k})}\right]+c\left({\hat{x}}^{k}\right)\log\left(2\beta \right)+\log\left\{2\beta \left[c\left({\hat{x}}^{k}\right)+1\right]\right\}\\ & \leq & c\left({\hat{x}}^{k}\right)\log c\left({\hat{x}}^{k}\right)+\log e+\frac{k(\log\beta )\log(2\beta )}{(1-\epsilon_{k})\log k}+\log\left[2\beta \left(k+1\right)\right]\\ & \overset{▵}{=} & c\left({\hat{x}}^{k}\right)\log c\left({\hat{x}}^{k}\right)+k\cdot \varepsilon \left(k\right), \end{array}$$

where we note that β is the cardinality of $\hat{X}$, and where $\epsilon_{k}$ and ε(k) tend to zero as k→∞.

Our first lower bound is given in the following theorem.

**Theorem** **1.** *Consider the setting formulated in Section 2. Then, for every $x^{n}\in X^{n}$,*

(9) $$\rho \left(x^{n};E\left(q,k,D\right)\right)\geq \frac{1}{n}\sum_{i=0}^{n/k-1}\min_{{\hat{x}}^{k}\in B\left(x_{ik+1}^{ik+k},D\right)}c\left({\hat{x}}^{k}\right)\log c\left({\hat{x}}^{k}\right)-\frac{\left(\log\beta \right)\log\left(4q^{2}\right)}{(1-\epsilon_{k})\log k}-\frac{q^{2}\log\left(4q^{2}\right)}{k}.$$

**Proof** **of** **Theorem** **1.** The proof is conceptually simple. Since each *k*-block, ${\hat{x}}_{ik+1}^{ik+k}$, i=0,1,…,n/k−1, of the reconstruction vector, ${\hat{x}}^{n}$, is compressed using a finite-state machine with *q* states, then, according to [27] Theorem 1, its compression ratio is lower bounded by

(10) $$\begin{array}{ccc} \frac{c\left({\hat{x}}_{ik+1}^{ik+k}\right)+q^{2}}{k}\log\frac{c\left({\hat{x}}_{ik+1}^{ik+k}\right)+q^{2}}{4q^{2}} & \geq & \frac{c({\hat{x}}_{ik+1}^{ik+k})}{k}\log c\left({\hat{x}}_{ik+1}^{ik+k}\right)-\frac{c\left({\hat{x}}_{ik+1}^{ik+k}\right)+q^{2}}{k}\log\left(4q^{2}\right)\\ & \geq & \frac{c({\hat{x}}_{ik+1}^{ik+k})}{k}\log c\left({\hat{x}}_{ik+1}^{ik+k}\right)-\frac{\left(\log\beta \right)\log\left(4q^{2}\right)}{(1-\epsilon_{k})\log k}-\frac{q^{2}\log\left(4q^{2}\right)}{k}, \end{array}$$

where the second inequality follows from [27] Equation (6). Since each *k*-block must comply with the distortion constraint, this quantity is further lower bounded by

$$\min_{{\hat{x}}^{k}\in B\left(x_{ik+1}^{ik+k},D\right)}\frac{c({\hat{x}}^{k})}{k}\log c\left({\hat{x}}^{k}\right)-\frac{\left(\log\beta \right)\log\left(4q^{2}\right)}{(1-\epsilon_{k})\log k}-\frac{q^{2}\log\left(4q^{2}\right)}{k},$$

and so, for the entire source vector $x^{n}$, we have

(11) $$\rho \left(x^{n};E\left(q,k,D\right)\right)\geq \frac{1}{n}\sum_{i=0}^{n/k-1}\min_{{\hat{x}}^{k}\in B\left(x_{ik+1}^{ik+k},D\right)}c\left({\hat{x}}^{k}\right)\log c\left({\hat{x}}^{k}\right)-\frac{\left(\log\beta \right)\log\left(4q^{2}\right)}{(1-\epsilon_{k})\log k}-\frac{q^{2}\log\left(4q^{2}\right)}{k}.$$

This completes the proof of Theorem 1. □

For large enough *k*, the last two terms can be made arbitrarily small, provided that logq≪logk. Clearly, this lower bound can be asymptotically attained by seeking the vector ${\hat{x}}^{k}\in \hat{X^{k}}$ that minimizes $c\left({\hat{x}}^{k}\right)\log c\left({\hat{x}}^{k}\right)$ across $B(x_{ik+1}^{ik+k},D)$ within each *k*-block and compressing it by the LZ78 compression algorithm.

In order to state our second lower bound, we next define the joint empirical distribution of *ℓ*-blocks of ${\hat{x}}_{ik+1}^{ik+k}$. Specifically, let *ℓ* divide *k*, which in turn divides *n*, and consider the empirical distribution, ${\hat{P}}_{i}=\left\{{\hat{P}}_{i}\left({\hat{x}}^{\ell }\right),\;{\hat{x}}^{\ell }\in {\hat{X}}^{\ell }\right\}$, of *ℓ*-vectors along the *i*-th *k*-block of ${\hat{x}}^{n}$, which is ${\hat{x}}_{ik+1}^{ik+k}$, i=0,1,…,n/k−1, that is,

(12) $${\hat{P}}_{i}\left({\hat{x}}^{\ell }\right)=\frac{\ell }{k}\sum_{j=0}^{k/\ell -1}I\left\{{\hat{x}}_{ik+j\ell +1}^{ik+j\ell +\ell }={\hat{x}}^{\ell }\right\},\;\;\;\;{\hat{x}}^{\ell }\in {\hat{X}}^{\ell }.$$

Let $\hat{H}\left({\hat{X}}_{i}^{\ell }\right)$ denote the empirical entropy of an auxiliary random *ℓ*-vector, ${\hat{X}}_{i}^{\ell }$, induced by ${\hat{P}}_{i}$, that is,

(13) $$\hat{H}\left({\hat{X}}_{i}^{\ell }\right)=-\sum_{{\hat{x}}^{\ell }\in {\hat{X}}^{\ell }}{\hat{P}}_{i}\left({\hat{x}}^{\ell }\right)\log{\hat{P}}_{i}\left({\hat{x}}^{\ell }\right).$$

Now, our second lower bound is given in the following theorem.

**Theorem** **2.** *Consider the setting formulated in Section 2. Then, for every $x^{n}\in X^{n}$,*

(14) $$\rho \left(x^{n};E\left(q,k,D\right)\right)\geq \frac{k}{n}\sum_{i=0}^{n/k-1}\min_{{\hat{x}}_{ik+1}^{ik+k}\in B\left(x_{ik+1}^{ik+k},D\right)}\frac{\hat{H}\left({\hat{X}}_{i}^{\ell }\right)}{\ell }-\frac{1}{\ell }\log\left\{q^{2}\left(1+\log\left[1+\frac{\beta^{\ell }}{q^{2}}\right]\right)\right\}.$$

**Discussion.**

Note that both lower bounds depend on the number of states, *q*, of the FSLE, but not on the number of states, |S|, of the FSRE. In this sense, no matter how large the number of states of the FSRE may be, none of these bounds is affected. For the purpose of lower bounds, which establish fundamental limitations, we wish to consider a class of encoders that is as broad as possible, for the sake of generality. Therefore, we assume that S is arbitrarily large.

The second term on the right-hand side of (14) is small when logq is small, relative to *ℓ*, which is in turn smaller than *k*. This requirement is less restrictive than the parallel one in the first bound, which was logq≪logk. The bound is asymptotically achievable by the universal lossless coding of the vector ${\hat{x}}_{ik+1}^{ik+k}$ that minimizes $\hat{H}\left({\hat{X}}_{i}^{\ell }\right)$ within $B(x_{ik+1}^{ik+k},D)$ using a universal lossless code that is based on two-part coding: the first part is a header that indicates the type class ${\hat{P}}_{i}$ using a logarithmic number of bits as a function of *k* and the second part is the index of the vector within the type class.

The main term of the second bound is essentially tighter than the main term of the first bound since $\hat{H}\left({\hat{X}}_{i}^{\ell }\right)$ can be lower bounded by $c\left({\hat{x}}_{ik+1}^{ik+k}\right)\log c\left({\hat{x}}_{ik+1}^{ik+k}\right)$, minus some small terms (see, e.g., [35] Equation (26)). On the other hand, the second bound is somewhat more complicated due to the introduction of the additional parameter *ℓ*. It is not clear whether any one of the bounds completely dominates the other one for any $x^{n}$. It is always possible to choose the larger bound between the two.

**Proof** **of** **Theorem** **2.** According to [27] Lemma 2, since the FSLE is an information lossless encoder with *q* states, it must obey the following generalized Kraft inequality:

(15) $$\sum_{{\hat{x}}^{\ell }\in {\hat{X}}^{\ell }}2^{-\min_{z\in Z}L\left[f\left(z,{\hat{x}}^{\ell }\right)\right]}\leq q^{2}\left(1+\log\left[1+\frac{\beta^{\ell }}{q^{2}}\right]\right).$$

This implies that the description length at the output of the encoder is lower bounded as follows:

(16) $$\begin{array}{ccc} L(b^{n}) & = & \sum_{t=1}^{n}L\left[f\left(z_{t},{\hat{x}}_{t}\right)\right]\\ & = & \sum_{i=0}^{n/k-1}\sum_{m=0}^{k/\ell -1}\sum_{j=1}^{\ell }L\left[f\left(z_{ik+m\ell +j},{\hat{x}}_{ik+m\ell +j}\right)\right]\\ & = & \sum_{i=0}^{n/k-1}\sum_{m=0}^{k/\ell -1}L\left[f\left(z_{ik+m\ell +1},{\hat{x}}_{ik+m\ell +1}^{ik+m\ell +\ell }\right)\right]\\ & \geq & \sum_{i=0}^{n/k-1}\sum_{m=0}^{k/\ell -1}\min_{z\in Z}L\left[f\left(z,{\hat{x}}_{ik+m\ell +1}^{ik+m\ell +\ell }\right)\right]\\ & = & \sum_{i=0}^{n/k-1}\frac{k}{\ell }\sum_{{\hat{x}}^{\ell }\in {\hat{X}}^{\ell }}{\hat{P}}_{i}\left({\hat{x}}^{\ell }\right)\cdot \min_{z\in Z}L\left[f\left(z,{\hat{x}}^{\ell }\right)\right], \end{array}$$

Clearly,

(17) $$\frac{L(b^{n})}{n}\geq \frac{k}{n}\sum_{i=0}^{n/k-1}\frac{1}{\ell }\sum_{{\hat{x}}^{\ell }\in {\hat{X}}^{\ell }}{\hat{P}}_{i}\left({\hat{x}}^{\ell }\right)\cdot \min_{z\in Z}L\left[f\left(z,{\hat{x}}^{\ell }\right)\right].$$

Now, by the generalized Kraft inequality above,

(18) $$\begin{array}{ccc} q^{2}\left(1+\log\left[1+\frac{\beta^{\ell }}{q^{2}}\right]\right) & \geq & \sum_{{\hat{x}}^{\ell }\in {\hat{X}}^{\ell }}2^{-\min_{z\in Z}L\left[f\left(z,{\hat{x}}^{\ell }\right)\right]}\\ & \geq & \sum_{{\hat{x}}^{\ell }\in {\hat{X}}^{\ell }}{\hat{P}}_{i}\left({\hat{x}}^{\ell }\right)\cdot 2^{-\min_{z\in Z}L[f\left(z,{\hat{x}}^{\ell }\right)-\log{\hat{P}}_{i}\left({\hat{x}}^{\ell }\right)}\\ & \geq & \exp_{2}\left\{-\sum_{{\hat{x}}^{\ell }\in {\hat{X}}^{\ell }}{\hat{P}}_{i}\left({\hat{x}}^{\ell }\right)\cdot \min_{z\in Z}L[f\left(z,{\hat{x}}^{\ell }\right)+\hat{H}\left({\hat{X}}_{i}^{\ell }\right)\right\}, \end{array}$$

where the last inequality follows from the convexity of the exponential function and Jensen’s inequality. This yields

(19) $$\log\left\{q^{2}\left(1+\log\left[1+\frac{\beta^{\ell }}{q^{2}}\right]\right)\right\}\geq \hat{H}\left({\hat{X}}_{i}^{\ell }\right)-\sum_{{\hat{x}}^{\ell }\in {\hat{X}}^{\ell }}{\hat{P}}_{i}\left({\hat{x}}^{\ell }\right)\cdot \min_{z\in Z}L\left[f\left(z,{\hat{x}}^{\ell }\right)\right],$$

implying that

(20) $$\begin{array}{ccc} \frac{L(b^{n})}{n} & \geq & \frac{k}{n}\sum_{i=0}^{n/k-1}\frac{1}{\ell }\sum_{{\hat{x}}^{\ell }\in {\hat{X}}^{\ell }}{\hat{P}}_{i}\left({\hat{x}}^{\ell }\right)\cdot \min_{z\in Z}L\left[f\left(z,{\hat{x}}^{\ell }\right)\right]\\ & \geq & \frac{k}{n}\sum_{i=0}^{n/k-1}\frac{\hat{H}\left({\hat{X}}_{i}^{\ell }\right)}{\ell }-\frac{1}{\ell }\log\left\{q^{2}\left(1+\log\left[1+\frac{\beta^{\ell }}{q^{2}}\right]\right)\right\}, \end{array}$$

and since each ${\hat{x}}_{ik+1}^{ik+k}$ must be in $B(x_{ik+1}^{ik+k}),D)$, the summand of the first term on the left-hand side cannot be smaller than $\min_{{\hat{x}}^{k}\in B\left(x_{ik+1}^{ik+k}\right),D)}\hat{H}\left({\hat{X}}_{i}^{\ell }\right)/\ell$. Since this lower bound on $L(b^{n})/n$ holds for every E∈E(q,k,D), it holds also for $\rho (x^{n};E\left(q,k,D\right))$. This completes the proof of Theorem 2. □

Returning now to the first lower bound, consider the following chain of inequalities:

(21) $$\begin{array}{ccc} \sum_{i=0}^{n/k-1}\min_{{\hat{x}}^{k}\in B\left(x_{ik+1}^{ik+k},D\right)}c\left({\hat{x}}^{k}\right)\log c\left({\hat{x}}^{k}\right) & \geq & \sum_{i=0}^{n/k-1}\min_{{\hat{x}}^{k}\in B\left(x_{ik+1}^{ik+k},D\right)}LZ\left({\hat{x}}^{k}\right)-k\varepsilon \left(k\right)\\ & = & -\sum_{i=0}^{n/k-1}\log\left[\max_{{\hat{x}}^{k}\in B\left(x_{ik+1}^{ik+k},D\right)}2^{-LZ({\hat{x}}^{k})}\right]-k\varepsilon \left(k\right)\\ & \geq & -\sum_{i=0}^{n/k-1}\log\left[\sum_{{\hat{x}}^{k}\in B\left(x_{ik+1}^{ik+k},D\right)}2^{-LZ({\hat{x}}^{k})}\right]-k\varepsilon \left(k\right). \end{array}$$

It is conceivable that the last inequality may contribute to most of the gap between the left-most side and the right-most side of the chain (21), since we pass from a single term in $B(x_{ik+1}^{ik+k},D)$ to the sum of all terms in $B(x_{ik+1}^{ik+k},D)$. Since

(22) $$\max_{{\hat{x}}^{k}\in B\left(x_{ik+1}^{ik+k},D\right)}2^{-LZ({\hat{x}}^{k})}\leq \sum_{{\hat{x}}^{k}\in B\left(x_{ik+1}^{ik+k},D\right)}2^{-LZ({\hat{x}}^{k})}\leq \left|B\left(x_{ik+1}^{ik+k},D\right)\right|\cdot \max_{{\hat{x}}^{k}\in B\left(x_{ik+1}^{ik+k},D\right)}2^{-LZ({\hat{x}}^{k})},$$

the gap between the left-most side of (21) and the right-most side of (21) might take any positive value that does not exceed $\log|B(\left(x_{ik+1}^{ik+k},D\right)|$, which is in turn approximately proportional to *k* as $\left|B(x_{ik+1}^{ik+k},D)\right|$ is asymptotically exponential in *k*. Thus, the right-most side of (21), corresponds to a coding rate, which might be strictly smaller than that of the left-most side. Yet, we argue that the right-most side of (21) can still be asymptotically attained by a finite-state encoder. But to this end, its FSLE component should possess $q=\beta^{k}$ states, as it is actually a block code of length *k*. In order to see this, we need to define the following *universal probability distribution* (see also [35] and references therein):

(23) $$U\left({\hat{x}}^{k}\right)=\frac{2^{-LZ({\hat{x}}^{k})}}{\sum_{{\tilde{x}}^{k}\in {\hat{X}}^{k}}2^{-LZ({\tilde{x}}^{k})}}\overset{▵}{=}\frac{2^{-LZ({\hat{x}}^{k})}}{Z},\;\;\;\;{\hat{x}}^{k}\in {\hat{X}}^{k},$$

and accordingly, also define

(24) $$U\left[B\left(x^{k},D\right)\right]=\sum_{{\hat{x}}^{k}\in B\left(x^{k},D\right)}U\left({\hat{x}}^{k}\right).$$

Now, the first term on the right-most side of (21) can be further manipulated as follows:

(25) $$\begin{array}{ccc} -\sum_{i=0}^{n/k-1}\log\left[\sum_{{\hat{x}}^{k}\in B\left(x_{ik+1}^{ik+k},D\right)}2^{-LZ({\hat{x}}^{k})}\right] & = & -\sum_{i=0}^{n/k-1}\log\left[\sum_{{\hat{x}}^{k}\in B\left(x_{ik+1}^{ik+k},D\right)}U\left({\hat{x}}^{k}\right)\cdot Z\right]\\ & \geq & -\sum_{i=0}^{n/k-1}\log U\left[B\left(x_{ik+1}^{ik+k},D\right)\right], \end{array}$$

where the last inequality is due to the fact that −logZ≥0, thanks to Kraft’s inequality applied to the code-length function LZ(·).

Now, the last expression in (25) suggests achievability using the universal distribution, *U*, for the independent random selection of various codewords. The basic idea is quite standard and simple: The quantity, $U[B\left(x_{ik+1}^{ik+k},D\right)]$, is the probability that a single randomly chosen reproduction vector, drawn under *U*, would fall within distance kD from the source vector, $x_{ik+1}^{ik+k}$. If all reproduction codewords are drawn independently under *U*, then the typical number of random selections required before one sees the first one in $B(x_{ik+1}^{ik+k},D)$ is of the exponential order of $1/U[B\left(x_{ik+1}^{ik+k},D\right)]$. Given that the codebook is revealed to both the encoder and decoder, once it has been selected, the encoder merely needs to transmit the index of the first reproduction vector within the codebook, and the description length of that index can be made essentially as small as $\log\left\{1/U\left[B\left(x_{ik+1}^{ik+k},D\right)\right]\right\}=-\log\left(U\left[B\left(x_{ik+1}^{ik+k},D\right)\right]\right)$. In [35], we use this simple idea to prove achievability for an arbitrary distortion measure. More precisely, the following theorem is stated and proved in [35] with some adjustments to the notation:

**Theorem** **3** ([35] Theorem 2). *Let $d:X^{k}\times {\hat{X}}^{k}\to R^{+}$ be an arbitrary distortion function. Then, for every ϵ>0, there exists a sequence of d-semifaithful, variable-length block codes of block length k, such that for every $x^{k}\in X^{k}$, the code length for $x^{k}$ is upper bounded by*

(26) $$L\left(x_{ik+1}^{ik+k}\right)\leq -\log\left(U\left[B\left(x_{ik+1}^{ik+k},D\right)\right]\right)+\left(2+\epsilon \right)\log k+c+\delta_{k},$$

*where c>0 is a constant and $\delta_{k}=O\left(k\beta^{k}e^{-k^{1+\epsilon }}\right)$.*

Through the repeated application of this code for each one of the n/k blocks of length *k*, the lower bound of the last line of (25) is asymptotically attained. As elaborated on in [35], the ensemble of codebooks selected under the universal distribution, *U*, exhibits universality in both the source sequence slated for encoding and the chosen distortion measure. This stands in contrast to the classical random coding distribution, which typically relies on both the statistics of the source and the characteristics of the distortion measure.

**Discussion.**

A natural question that may arise is whether this performance is the best that can be attained given that the number of FSLE states, *q*, is as large as $\beta^{k}$. For now, this question remains open, but it is conjectured that the answer is affirmative, in view of the matching converse theorem of [35] Theorem 1, which applies to the vast majority of source sequences in every type class of any order, even without the limitation of finite-state encoders.

It is natural to think of the memory resource used by a finite-state encoder in terms of the number of bits (or equivalently, the size of a register) needed in order to store the current state at each time instant, namely, the base 2 logarithm of the total number of states. Indeed, both lower bounds derived earlier contain terms that are proportional to logq, the memory size pertaining to the FSLE. Since the memory size, log|S|, of the FSRE is assumed arbitrarily large, as discussed earlier, the total size of the encoder memory, log|S|+logq, is dominated by log|S|, and so, the contribution of logq to the total memory volume can be considered negligibly small. Therefore, one of our main messages in this work is that, as far as the total memory size goes, it makes very little difference if we allow logq to be as large as klogβ and, thereby, achieve better performance, rather than keeping logq smaller and ending up with the inferior compression performance of minimizing $LZ({\hat{x}}_{ik+1}^{ik+k})$ within $B(x_{ik+1}^{ik+k},D)$ for each block.

## 4. Conclusions

In this paper, we revisited the paradigm of lossy compression of individual sequences using finite-state machines, as a natural extension of the same paradigm in the lossless case, as established by Ziv and Lempel in [27] and other related works. This work can also be viewed as a revisit of [35] from the perspective of finite-state encoding of individual sequences. Our model of a finite-state encoder is that of a cascade of the finite-state *k*-dimensional reproduction encoder (with an arbitrarily large number of states) and a finite-state lossless encoder, acting on the reproduction sequence. Our main contributions to this work are as follows:

1. We proposed a model of a finite-state lossy encoder, composed of a cascade of an FSRE and an FSLE.
2. We derived two different lower bounds to the compression ratio.
3. We showed that both bounds depend on the number of states, *q*, of the lossless encoder, but not on the number of states of the reproduction encoder.
4. We showed that for relatively small *q*, one cannot do better than seeking the most compressible reproduction sequence within the ’sphere’ of radius, kD, around the source vector. Nonetheless, if we allow $q=\beta^{k}$, we can improve performance significantly by using a good code from the ensemble of codes, where each codeword is selected independently at random under the universal distribution, *U*. The resulting code is universal, not only in the sense of the source sequence, as in [27], but also in the distortion function, in the sense discussed in [35]. This passage from small *q* to large *q* will not increase the total memory resources of the entire encoder significantly, considering the large memory that may be used by the reproduction encoder anyway.
5. We suggested the conjecture that the performance achieved, as described in item 3, is the best performance achievable for large *q*.

Finally, our derivations can be extended to incorporate side information, $u^{n}=\left(u_{1},u_{2},\ldots ,u_{n}\right)$, available to both the encoder and decoder. In the model of the finite-state encoder, this amounts to allowing both the FSRE and the FSLE sequential access to $u_{t}$, t=1,2,…. The decoder, of course, should also have access to $u^{n}$. Another modification needed is to replace the LZ algorithm with its conditional version in all places (see, e.g., [31,39]). 

## Data Availability

Data are contained within the article.
